# Detailed genetic analysis of hemagglutinin-neuraminidase glycoprotein gene in human parainfluenza virus type 1 isolates from patients with acute respiratory infection between 2002 and 2009 in Yamagata prefecture, Japan

**DOI:** 10.1186/1743-422X-8-533

**Published:** 2011-12-13

**Authors:** Katsumi Mizuta, Mika Saitoh, Miho Kobayashi, Hiroyuki Tsukagoshi, Yoko Aoki, Tatsuya Ikeda, Chieko Abiko, Noriko Katsushima, Tsutomu Itagaki, Masahiro Noda, Kunihisa Kozawa, Tadayuki Ahiko, Hirokazu Kimura

**Affiliations:** 1 Yamagata Prefectural Institute of Public Health, 1-6-6 Toka-machi, Yamagata-shi, Yamagata 990-0031, Japan; 2 Gunma Prefectural Institute of Public Health and Environmental Sciences, 378 Kamioki-machi, Maebashi-shi, Gunma 371-0052, Japan; 3 Katsushima Pediatric Clinic, 4-4-12 Minamidate, Yamagata-shi, Yamagata 990-2461, Japan; 4 Yamanobe Pediatric Clinic, 2908-14 Yamanobe-machi, Higashimurayama-gun, Yamagata 990-0301, Japan; 5 Department of Virology III, National Institute of Infectious Diseases, 4-7-1 Gakuen, Musashimurayama-shi, Tokyo 208-0011, Japan; 6 Infectious Disease Surveillance Center, National Institute of Infectious Diseases, 4-7-1 Gakuen, Musashimurayama-shi, Tokyo 208-0011, Japan

**Keywords:** Human parainfluenza virus, Maximum likelihood (ML) method, Phylogenetic analysis

## Abstract

**Background:**

Human parainfluenza virus type 1 (HPIV1) causes various acute respiratory infections (ARI). Hemagglutinin-neuraminidase (HN) glycoprotein of HPIV1 is a major antigen. However, the molecular epidemiology and genetic characteristics of such ARI are not exactly known. Recent studies suggested that a phylogenetic analysis tool, namely the maximum likelihood (ML) method, may be applied to estimate the evolutionary time scale of various viruses. Thus, we conducted detailed genetic analyses including homology analysis, phylogenetic analysis (using both the neighbor joining (NJ) and ML methods), and analysis of the pairwise distances of *HN *gene in HPIV1 isolated from patients with ARI in Yamagata prefecture, Japan.

**Results:**

A few substitutions of nucleotides in the second binding site of *HN *gene were observed among the present isolates. The strains were classified into two major clusters in the phylogenetic tree by the NJ method. Another phylogenetic tree constructed by the ML method showed that the strains diversified in the late 1980s. No positively selected sites were found in the present strains. Moreover, the pairwise distance among the present isolates was relatively short.

**Conclusions:**

The evolution of *HN *gene in the present HPIV1 isolates was relatively slow. The ML method may be a useful phylogenetic method to estimate the evolutionary time scale of HPIV and other viruses.

## Background

Human parainfluenza virus type 1 (HPIV1) of the genus *Respirovirus *and family *Paramyxoviridae *causes various acute respiratory infections (ARI) including the common cold, croup, bronchiolitis, and pneumonia [1]. Epidemiological data suggest that HPIV types 1-4 mainly infect younger children at least once, although reinfections may occur in adults [2,3]. Indeed, serological surveys indicate that at least 75% of children have been infected with HPIV1 by 5 years of age [4,5]. HPIV1 and 3 show high prevalence and are associated with up to 12% of acute lower respiratory tract infections in adults [6]. Thus, HPIVs, including HPIV1, may be major agents of ARI throughout the world [7-9].

HPIV possess two major surface glycoproteins: hemagglutinin-neuraminidase (HN) glycoprotein and fusion protein (F protein) [1]. HN glycoprotein shows multiple biological functions that include hemagglutinin and enzymatic activities as neuraminidase [3,10]. As a result, this molecule regulates viral adsorption and entry, and regulates the release of progeny virions from the infected cell surface [3]. In addition, it is suggested that HN glycoprotein is a major antigen [1]. The detailed molecular characteristics of HN glycoprotein have been confirmed in HPIV3, while those in HPIV1 remain unclear [11]. In addition, the genetic characteristics of HPIV1 are poorly understood. Thus, it is important to analyze the *HN *coding region in HPIV1.

The neighbor joining (NJ) method is frequently used in phylogenetic analysis to examine the molecular epidemiology of various viral genomes [12,13]. This method is based on a cluster classification algorithm, enabling the analysis of clusters and of the rate of viral evolution. Furthermore, the maximum likelihood (ML) method enables an estimation of the evolutionary time scale [14]. Using these methods, we conducted a detailed genetic analysis of the *HN *coding region in HPIV1 isolates from patients with ARI in Yamagata prefecture, Japan.

## Methods

### Patients and isolation of HPIV1

A total of 182 throat and nasal swab specimens were collected from patients attending pediatric clinics in Yamagata prefecture from May 2002 to November 2009. Informed consent was obtained from the parents of all subjects for the donation of samples used in this study. All patients were aged from 0 to 43 years (4.1 ± 5.0 years; mean ± SD). Patients were mainly diagnosed with upper respiratory illness (URI) and wheezy bronchiolitis (Additional file 1: Table S1). URI is also known as the common cold and typically affects the upper airways, including the nose (sinusitis), throat (pharyngitis), and larynx (laryngitis) [15]. Wheezy bronchiolitis was defined as the presence of wheezing alone or chest retractions in association with URI [16].

### Cell culture and virus isolation

In this study, human embryonic lung fibroblast (HEF), human laryngeal carcinoma (HEp-2), African green monkey kidney (Vero E6), Madin Darby canine kidney (MDCK), rhabdomyosarcoma (RD-18S), green monkey kidney (GMK), and human melanoma (HMV-II) cell lines were grown in Roswell Park Memorial Institute medium (Nissui No.3; Nissui Pharmaceutical Co., Ltd., Tokyo, Japan) containing 5-10% fetal bovine serum or calf serum at 37°C in a humidified atmosphere of 5% CO_2 _[17]. Each cell line was prepared in 96-well tissue culture plates (Greiner Bio-One, GmbH, Frickenhausen, Germany). We inoculated the throat and nasal swabs from patients onto the plates and incubated them at 33°C in a humidified atmosphere of 5% CO_2_. The cytopathic effects (CPEs) in the cells were observed 2-3 times per week. We harvested the culture supernatant when a suspected HPIV CPE was observed or a hemadsorption (HAD) test with 0.8% guinea pig erythrocytes was positive, and stored it at -80°C until analysis.

### RNA extraction, RT-PCR, and sequencing

Viral RNA of HPIV1 isolates was extracted from 200 μL of the viral culture fluid by using ISOGEN (Nippon Gene, Tokyo, Japan) and was then transcribed into cDNA with M-MLV reverse transcriptase (Nippon Gene) and a random primer (Takara Bio Inc., Otsu, Japan). Reverse transcription was carried out at 30°C for 10 min, followed by incubation at 37°C for 45 min, and then incubation at 95°C for 5 min. To amplify the *HN *coding region in HPIV1 gene (nucleotide position 7245-8477, 1233 nt) by RT-PCR, we designed new primer sets using Primer Express (R) version 1.5 software (Applied Biosystems LLC, Foster City, CA) [18]. Primer sequences were as follows: first primer pair, 5'- CAG AAT TAA TCA GAC AAG AAG TRA TAT CAA G -3' (primer HPIV-1USF, position 7165-7195), and 5'- TGA TAC GRA TTA AGA CAT TGA CAA CTT G -3' (primer HPIV-1USR, position 8034-8061); and second primer pair, 5'- CRA GTS RAG GWA TAG RAG AYT TAG TAT TTG -3'(primer HPIV-1DSF, position 7750-7779) and 5'- GGT TRA TTT CAA CAA TRT GGA AGC AGT A -3' (primer HPIV-1DSR, position 8529-8556). Nucleotide position was based on the sequence of *HN *glycoprotein genome in HPIV1 (Mil-48/91, GenBank accession no. U70936). Using cDNA solution, a DNA fragment of 1392 bp was amplified by PCR. Forty amplification cycles were performed at 94°C for 30 s, at 55°C for 30 s, at 72°C for 1 min, followed by a final extension at 72°C for 10 min. We analyzed the nucleotide sequences (1233b; located at 398-1633 within the *HN *gene) of the *HN *coding region. Briefly, the DNA fragment was purified with a QIAquick PCR Purification Kit (Qiagen, Valencia, CA), submitted to a cycle sequence with a Big Dye Terminator v3.1 Cycle Sequencing Kit (Applied Biosystems, Warrington, UK) using the two primer sets stated above, and purified with a spin column (AutoSeq G-50, Amersham Biosciences, Piscataway, NJ). The nucleotide sequence was determined with an automated DNA sequencer (ABI PRISM 3130 Genetic Analyzer, Applied Biosystems, Foster City, CA).

### Phylogenetic analysis and calculation of pairwise distances by the NJ method

Phylogenetic analysis of the nucleotide sequence of the *HN *coding region of HPIV1 was conducted with the CLUSTAL W program available from the DNA Data Bank of Japan http://www.ddbj.nig.ac.jp/index-j.html, and Tree Explorer version 2.12 [19].

Evolutionary distances were estimated according to Kimura's 2-parameter method, and the phylogenetic tree was constructed with the NJ method. The reliability of the tree was estimated with 1000 bootstrap replications. We used reference strains in this study to construct the phylogenetic tree. In addition, we calculated the pairwise distances for all strains, including the present isolates and reference strains to assess the frequency distribution among all HPIV1 strains and that of each intercluster of HPIV1, as previously described [20]. The GenBank accession numbers of the nucleotide sequences obtained in the present study are AB641132 to AB641313.

To evaluate the action of selective pressure on the *HN *coding regions among all HPIV1 strains, we estimated the rates of synonymous (dS) and non-synonymous (dN) changes at amino acid sites by conservative single likelihood ancestor counting (SLAC) and the fixed effects likelihood (FEL) method using ML available on the Datamonkey webserver http://www.datamonkey.org/[21]. The SLAC method is suitable for fast likelihood-based "counting methods" that employ either a single most likely ancestral reconstruction, weighted across all possible ancestral reconstructions, or sampling from ancestral reconstructions. The FEL method directly estimates dN and dS substitution rates at each site. These methods were performed to examine the dN and dS rates, incorporating the General Time Reversible (GTR) model of nucleotide substitution and the phylogenetic tree inferred using the NJ method. Positive (dN > dS) and negative (dN < dS) selections were predicted using these models. The *p*-value was used to classify a site as positively or negatively selected by these methods.

### Phylogenetic analysis and estimation of time scale by the ML method

To construct the phylogenetic tree by the ML method, which is the best nucleotide substitution model, the GTR with gamma distributed rates across sites (GTR + Γ) [22,23] was selected by the KAKUSAN4 program version 4.0. Supplementary materials related to this program can be found online at doi:10.1111/j.1755-0998.2011.03021.x. In this study, we used three phylogenetic models of sequence evolution. One is the different rate (DR) model [14], the most general, which assumes each branch of the unrooted phylogenetic tree has a different substitution rate [24]. The DR model is used as suitable general model against which to test the assumption of constant rates of the fit of the single rate (SR) and the single rate dated tips (SRDT) models [25]. Phylogenetic analysis of ML based on the DR model was constructed with RAxML BlackBox webserver http://phylobench.vital-it.ch/raxml-bb/index.php. All the present strains with more than 99.5% sequence similarity to any other strain were excluded from analysis. The reliability of the phylogenetic hypothesis was assessed using bootstrap analysis of 100 ML iterations. The SR model assumes the same rate of evolution in all branches (i.e., a molecular clock), and the SRDT model is an SR model that relaxes the assumption of contemporaneous sequences and uses the date of isolation of each sequence to estimate the substitution rate [24]. To estimate the rate of molecular evolution (and hence a time scale) for a phylogeny consisting of date tips, phylogenetic analyses by ML based on the SR and SRDT models were performed using the TipDate webserver http://mobyle.pasteur.fr/cgi-bin/portal.py?#forms::tipdate[24]. The likelihood of the SR and SRDT models (with likelihood L_0_) were compared to that of the DR model (with likelihood L_1_) in a likelihood ratio test (LRT) [14] of the fit of the model. The test statistic is the difference in the log likelihood (Δ) between the SR or SRDT model and the DR model. In the LRT statistic, twice the ratio of log likelihoods (L_1_/L_0_) (2Δ*) is expected to be *χ*^2 ^distributed with degrees of freedom equal to their models. The DR model has 2*n*-3 free parameters, the SR model has *n*-1, and the SRDT model has *n*-2 (a tree of *n *tips). In the LRT of the fit of the SR and SRDT models compared with the DR, if the SR model is rejected in favor of the DR model but the SRDT model is not, the SRDT model can be accepted as no worse a description of the evolution date than the DR model. A *P *value of ≥0.05 was considered statistically significant for the phylogenetic models. The resulting phylogenetic trees were described using TreeExplorer version 2.12. The rates of nucleotide substitution, the date estimating the root of the tree and the corresponding upper and lower 95% confidence intervals (CIs) were calculated under the SRDT model using the TipDate webserver.

## Results

### Phylogenetic analysis of the nucleotide sequences of the *HN *coding region in HPIV1 by the NJ method

**Figure 1:**
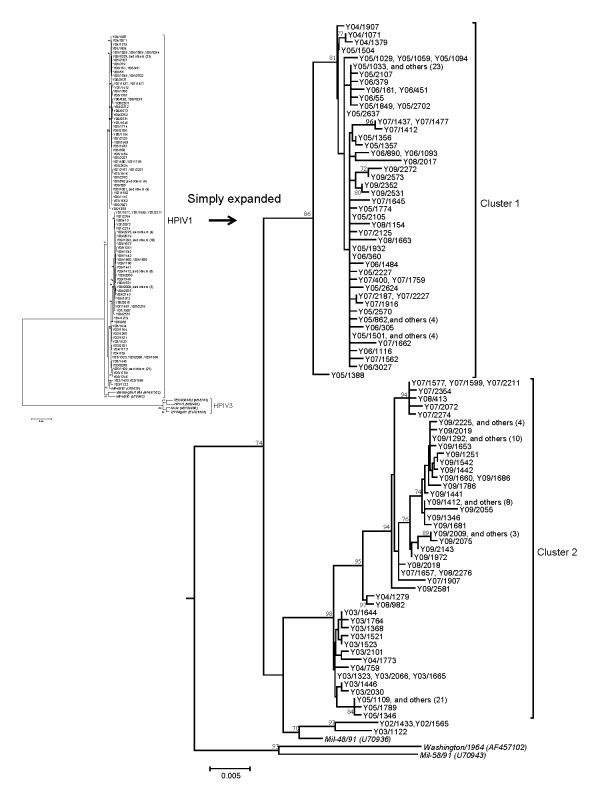
**Phylogenetic tree of HN region by NJ method**. Phylogenetic tree based on the nucleotide sequence of the *HN *coding region (1233nt), including the present strains (182) and representative reference strains (3). HPIV3 was used as an outgroup. Distance was calculated according to Kimura's 2-parameter method, and the tree was plotted with the neighbor-joining method. Reference strains are shown in italic type. The larger tree was simply expanded for HPIV1 strains, to clarify the distance of each of the minor clusters in the trees. The tree was constructed by neighbor-joining analysis with labeling of the branches showing at least 70% bootstrap support. The representation of the strain was changed from Yamagata/20XX/ZZZZ to YXX/ZZZZ.

The partial nucleotide sequences (1233 nt) of *HN *glycoprotein gene in a total of 182 isolates and 3 reference strains were analyzed. The phylogenetic tree based on the nucleotide sequences by the NJ method is shown in Figure 1. The enlarged cluster shows the HPIV1 strains. The phylogenetic tree containing the isolated and reference strains was classified into two unique clusters with the exception of three Yamagata strains (HPIVi/Yamagata/2002/1433, HPIVi/Yamagata/2002/1565, and HPIVi/Yamagata/2003/1122), clusters 1 and 2, and these strains were classified into different clusters from the reference strains. The number of detected strains in each cluster on the phylogenetic tree was as follows: cluster 1, 84 strains including strains isolated during 2004-2009; and cluster 2, 95 strains from 2003 to 2005, and 2007 to 2009.

### Likelihood ratio test (LRT) of the fit of the models and timescale evolution of the *HN *coding region in HPIV1 by the ML method

**Figure 2:**
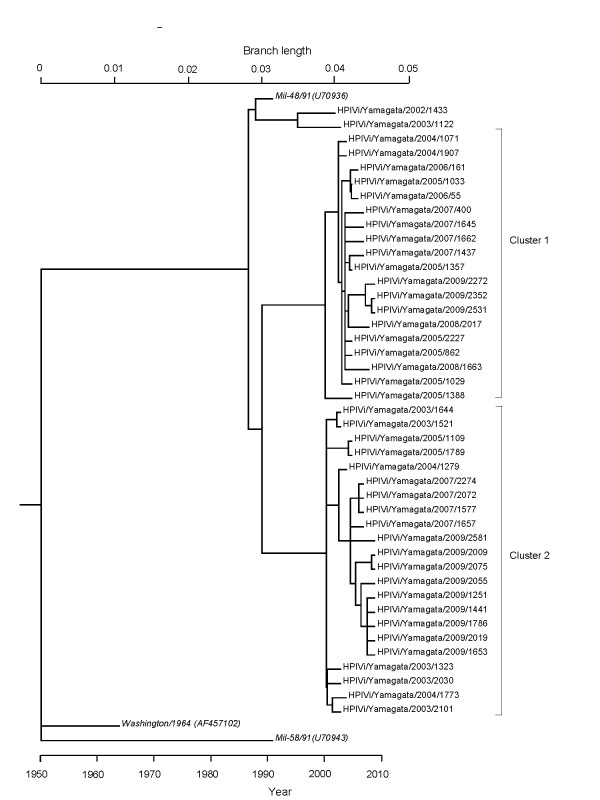
**Phylogenetic tree of HN region by ML method**. Phylogenetic tree based on the nucleotide sequence of the *HN *coding region (1233 nt), including the present strains (43) and representative reference strains (3), with branch lengths scaled under the SRDT model. Reference strains are shown in italic type.

Based on the SRDT model [24,26], we constructed another phylogenetic tree consisting of dated tips by using the nucleotide sequences of the HPIV1 *HN *coding region of 43 isolated strains from patients in Yamagata prefecture and 3 reference strains by using the ML method. As a result, the SR model was rejected as an adequate description of the evolution of the HPIV1 *HN *coding region (*p *< 0.05). Therefore, the SRDT model was not significantly worse than the DR model, indicating that this model adequately describes the substitution process (*p *= 0.06). Through these processes, we obtained a phylogenetic tree by the ML method. The year of the first major division in the present tree was estimated at 1950 (Figure 2; 95% CIs of 1935 to 1955). In addition, the ancestral strains of the present strains subdivided around 1987. Further division occurred around 1989, resulting in the formation of two major clusters (clusters 1 and 2). Furthermore, we estimated the rate of molecular evolution from the tree as 7.68 × 10^-4 ^substitutions per site per year (95% CIs of 4.80 × 10^-4 ^to 1.07 × 10^-3^).

### Analysis of pairwise distances, substitutions of the second binding site, and selective pressure of the *HN *coding region in HPIV1

**Figure 3:**
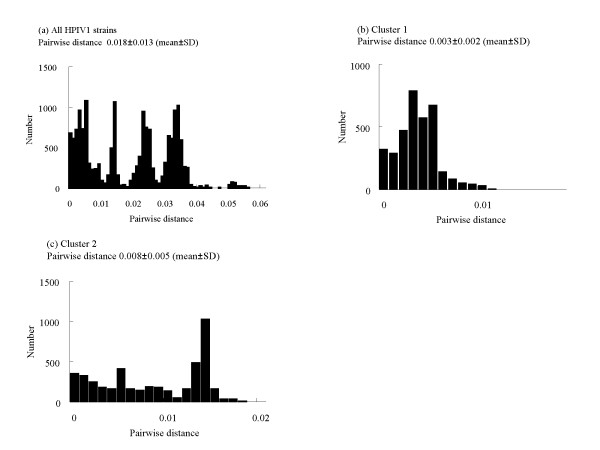
**Distributions of pairwise distances for HPIV1 of HN region**. (**a**) Distribution of pairwise distances for the 182 present and 3 reference strains. (**b**) Distribution of pairwise intercluster distances for cluster 1. (**c**) Distribution of pairwise intercluster distances for cluster 2.

The nucleotide and amino acid sequence identities among all 182 isolates were high at 92.6-100% and 96.0-100%, respectively. In addition, we calculated the intercluster distances of HPIV1 from the distribution of the pairwise distances. Based on the nucleotide sequences, the pairwise distance was 0.018 ± 0.013 [mean ± SD, Figure 3a] for the 182 present and 3 reference strains. The pairwise intercluster distances were as follows: cluster 1, 0.003 ± 0.002 (Figure 3b); and cluster 2, 0.008 ± 0.005 (Figure 3c). Cluster 2 showed a large pairwise intercluster distance, whereas that for cluster 1 was small. However, irregular peaks of pairwise distance are seen in Figure 3, and we were not able to genotyping the present strains based on the pairwise distance value. Next, amino acid substitutions in the analyzed *HN *coding region in the present strains were found at 31sites (detailed substitution data not shown). Among these was an essential substitution of the second binding site, N523S. This substitution (N523S) was seen in seven of the present strains (HPIVi/Yamagata/2007/1577, HPIVi/Yamagata/2007/1599, HPIVi/Yamagata/2007/2211, HPIVi/Yamagata/2007/2072, HPIVi/Yamagata/2007/2354, HPIVi/Yamagata/2007/2274, and HPIVi/Yamagata/2008/413). These strains belonged in cluster 2 (Figure 1).

Selection pressure analysis was performed in the present strains, and the results showed a low mean dN/dS ratio (0.17) (95% likelihood profile-based CIs, CI = 0.13-0.23) by the SLAC method. Thus, the nucleotide substitutions predicted were largely synonymous. The dN/dS ratios of the individual sites in the *HN *coding region were calculated by the SLAC and FEL methods significant at the *p *< 0.1 level. However, neither method detected a positively selected site.

## Discussion

In this study, we performed a detailed genetic analysis of *HN *glycoprotein gene in HPIV1 isolates from patients with ARI during 2002-2009 in Yamagata prefecture, Japan. The phylogenetic tree constructed by the NJ method showed that the present HPIV1 isolates were divisible into two major genetic clusters (Figure 1). The other tree constructed by the ML method showed that the year of the first major division was estimated at 1950, and the ancestral strains further subdivided at around 1987, resulting in three clusters (one minor and two major, Figure 2). The strains belonging to the two major clusters subdivided into many clusters after 2000. The present HPIV1 isolates showed an overall high level of nucleotide sequence identity (92.6-100%) of the *HN *coding region. Pairwise distance values based on the nucleotide sequences among the present strains were relatively low (less than 0.06). In addition, there were no positively selected sites found. These results suggest that several lineages of highly conserved *HN *gene in HPIV1 were prevalent in Yamagata prefecture. The present strains could not be provisionally type assigned from the pairwise distance values. Thus, the accumulation of large amounts of data may be needed to genotype HPIV1 based on pairwise distance.

Homology and phylogenetic analysis by the NJ method is frequently used in molecular epidemiological studies of various viruses. Homology analysis based on nucleotide sequences mainly shows the similarities of the analyzed genes among the strains. Phylogenetic analysis by the NJ method can give an estimation of the viral evolution rate and cluster classification. Furthermore, the ML method can enable analysis of the time scale of the evolution of viral genes. In the present study, we were able to estimate the viral evolution rate of HPIV1, cluster classification, and the evolutionary time scale of the present isolates by applying the NJ and ML methods to the detailed phylogenetic analysis of the *HN *coding region in HPIV1. There is currently little information available regarding the molecular evolution of the *HN *coding region in HPIV (HPIV1 to 4). Furthermore, the rate of molecular evolution is very low (7.68 × 10^-4 ^substitutions/site/year) in the present strains. Previous reports suggest that the rate of another gene of respiratory viruses belonging to *Paramyxoviridae*, such as respiratory syncytial (RS) virus, is higher (1.8 × 10^-3 ^substitutions/site/year) than that of the present data [27]. The reason for the difference is unknown at present. However, it is possible that genome properties other than size, such as polarity or structure, may be associated with substitutions of the viral genome [27]. Further studies on the detailed mechanisms of viral genome substitution may be needed. Additional sequence data and further structural analysis are required to demonstrate the mechanisms of the molecular evolution of HPIV1.

HPIV is classified into four serotypes (HPIV1 to 4), all of which can cause various ARI in humans, such as URI, croup, bronchitis, and pneumonia [1]. Previous reports suggest that HPIV1 and 3 are the dominant viruses in children with ARI [28]. In addition, HPIV is a major causative agent of virus-induced asthma [29]. Thus, HPIV1 is a major agent of ARI, along with other viruses, such as adenovirus, RS virus, human metapneumovirus, and rhinovirus [30]. However, the molecular epidemiology of HPIVs is poorly understood, and only a few reports on the molecular epidemiology of HPIV1 are available. For example, Henrickson and Savatski analyzed the longitudinal evolution of the *HN *coding region in 13 strains of HPIV1 isolated in the United States [31]. The results showed that the antigenic and genetic subgroups are very stable. Another report suggested that two distinct genotypes of HPIV were detected during the 1991 Milwaukee epidemic [32]. In the present study, we used HPIV1 isolates from patients with ARI and studied the evolution of HN protein, based on phylogenetic analyses using both the ML and NJ methods and the rate of the substitutions of nucleotides. The results showed that HN protein is highly conserved. In addition, no positively selected sites were detected. To our best knowledge, this is the first report of these findings in HPIV1.

The distribution of amino acids affects the structure of the *HN *coding region in HPIV1, and previous reports show that substitutions of amino acids in HN glycoprotein reveal second receptor binding sites [33,34]. For example, substitutions at Asn173 and Asn523 are critical for the formation of a second binding site. In particular, these substitutions affect, for example, the inhibitor in hemagglutination inhibition (HI) assays and infection of culture cells. However, the second receptor binding site did not significantly affect the growth or fusion activity of HPIV1. Substitutions at N523S were found in seven of the present strains, but there were no substitutions at Asn173. Thus, we thought that N523S may not be significantly associated with infectivity or pathogenicity.

Furthermore, we then examined selective pressure by counting and the ML method. Analysis of selection pressure in the present strains showed that dS substitutions predominated over dN substitutions, and no positively selected sites (substitution) were found in HN protein in the present HPIV1 strains. The evolution of the present strains may be largely driven by purifying selection. Compared with HPIV3, little is known about the detailed biological properties of HN glycoprotein in HPIV1. As an essential molecule of these viruses, further analysis of the biological properties of HN glycoprotein in HPIV1 is required [35].

Although detailed data of the antigenic and catalytic sites of HN molecules in HPIV3 is relatively clear [36], such information regarding HPIV1 is not yet known. Moreover, the epidemiology and molecular epidemiology of HPIV is not exactly known. Thus, further and larger epidemiological/molecular epidemiological studies are required to give better understanding of the etiology of HPIVs, including HPIV1.

## Conclusions

Our study suggested that several prevalent lineages of *HN *gene are highly conserved and evolution occurred from the late 1980s in HPIV1 in Yamagata prefecture. Viral evolution was estimated by detailed phylogenetic analyses using the NJ and ML methods. These methods offer a new understanding of the genetic evolution of *HN *gene.

## Abbreviations

HPIV1: Human parainfluenza virus type 1; ARI: Acute respiratory infections; HN: Hemagglutinin-neuraminidase; NJ: Neighbor joining; ML: Maximum likelihood; URI: Upper respiratory illness; CPE: Cytopathic effect; HAD: Hemadsorption; dS: Synonymous; dN: Non-synonymous; SLAC: Single likelihood ancestor counting; FEL: Fixed effects likelihood; GTR: General time reversible; DR: Different rate; SR: Single rate; SRDT: Single rate dated tips; LRT: Likelihood ratio test; CI: Confidence interval; RS: Respiratory syncytial; HI: Hemagglutination inhibition.

## Competing interests

The authors declare that they have no competing interests.

## Authors' contributions

KM and HK designed the research project; MS, MK, HT, YA, TI, CA, and NK performed the research; TI, MN, KK, and TA contributed the analytical tools; KM, MS, MK, and HK analyzed the data, and KM, MS, and HK wrote the paper. All authors read and approved the final manuscript.

## Supplementary Material

Additional file 1 **Table S1**. Subject data in this study. Click here for file
