# Supplementary material for: Detailed genetic analysis of hemagglutinin-neuraminidase glycoprotein gene in human parainfluenza virus type 1 isolates from patients with acute respiratory infection between 2002 and 2009 in Yamagata prefecture, Japan
Source: Virol J. 2011 Dec 13;8:533. doi: 10.1186/1743-422X-8-533 (PMC3295729; doi:10.1186/1743-422X-8-533)
Supplement: Additional file 1 — Table S1. Subject data in this study. [file 1743-422X-8-533-S1.DOC]

| Year | Clinical symptom | | | | | | Subtotal |
| --- | --- | --- | --- | --- | --- | --- | --- |
|  | URI | Wheezy bronchiolitis | Pneumonia | ILI | Others | Unknown |  |
| 2002 | 2 |  |  |  |  |  | 2 |
| 2003 | 9 | 1 | 1 | 1 |  |  | 12 |
| 2004 | 6 |  |  |  |  |  | 6 |
| 2005 | 60 | 9 | 2 | 1 |  |  | 72 |
| 2006 | 13 |  |  | 1 |  |  | 14 |
| 2007 | 19 | 1 |  |  |  |  | 20 |
| 2008 | 6 | 1 |  |  |  |  | 7 |
| 2009 | 41 | 2 | 1 |  | 2* | 3 | 49 |
| Subtotal | 156 | 14 | 4 | 3 | 2 | 3 | 182 |

Additional file 1: Table S1 Subject data in this study

URI:Upper respiratory illness

ILI:Influenza-like illness

*Influenza 1, pseudocroup 1
